# Supplementary material for: Lipofuscin-like autofluorescence within microglia and its impact on studying microglial engulfment
Source: Nat Commun. 2023 Nov 3;14:7060. doi: 10.1038/s41467-023-42809-y (PMC10624656; doi:10.1038/s41467-023-42809-y)
Supplement: Supplementary file 1 — Supplementary Information [file 41467_2023_42809_MOESM1_ESM.pdf]

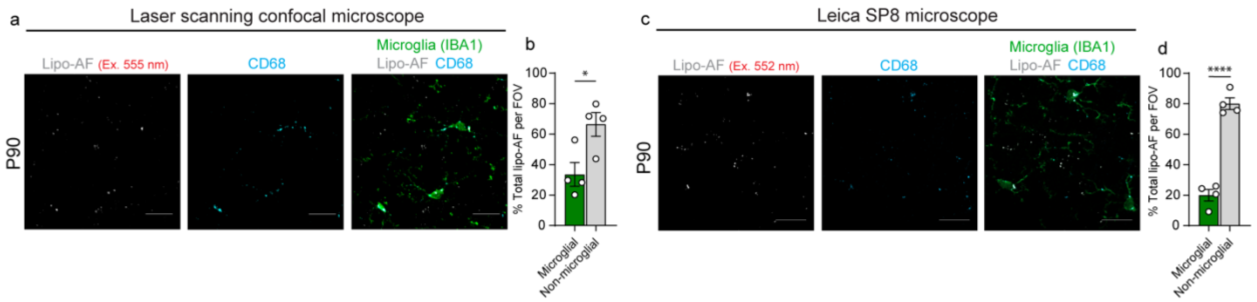


**Supplementary Figure 1. Microglial lipo-AF can be observed on different microscopes.**
**a,c** Representative images of an anti-IBA1 immunolabelled microglia in the P90 mouse somatosensory cortex containing lipo-AF within anti-CD68+ lysosomal compartments taken with a Zeiss laser scanning confocal microscope (a) or a Leica SP8 confocal microscope (c). Scale bars = 20 µm. **b,d** Quantification of the percentage of lipo-AF per field of view inside and outside of microglia. Data are represented as mean ± SEM, n = 4 mice. b Two-tailed unpaired t-test ($t = 2.990$, $df = 6$, $p=0.0243$); *$p<0.05$ and d ($t = 11.26$, $df = 6$, $p<0.0001$); ****$p<0.0001$. (3M, 1F for both systems).

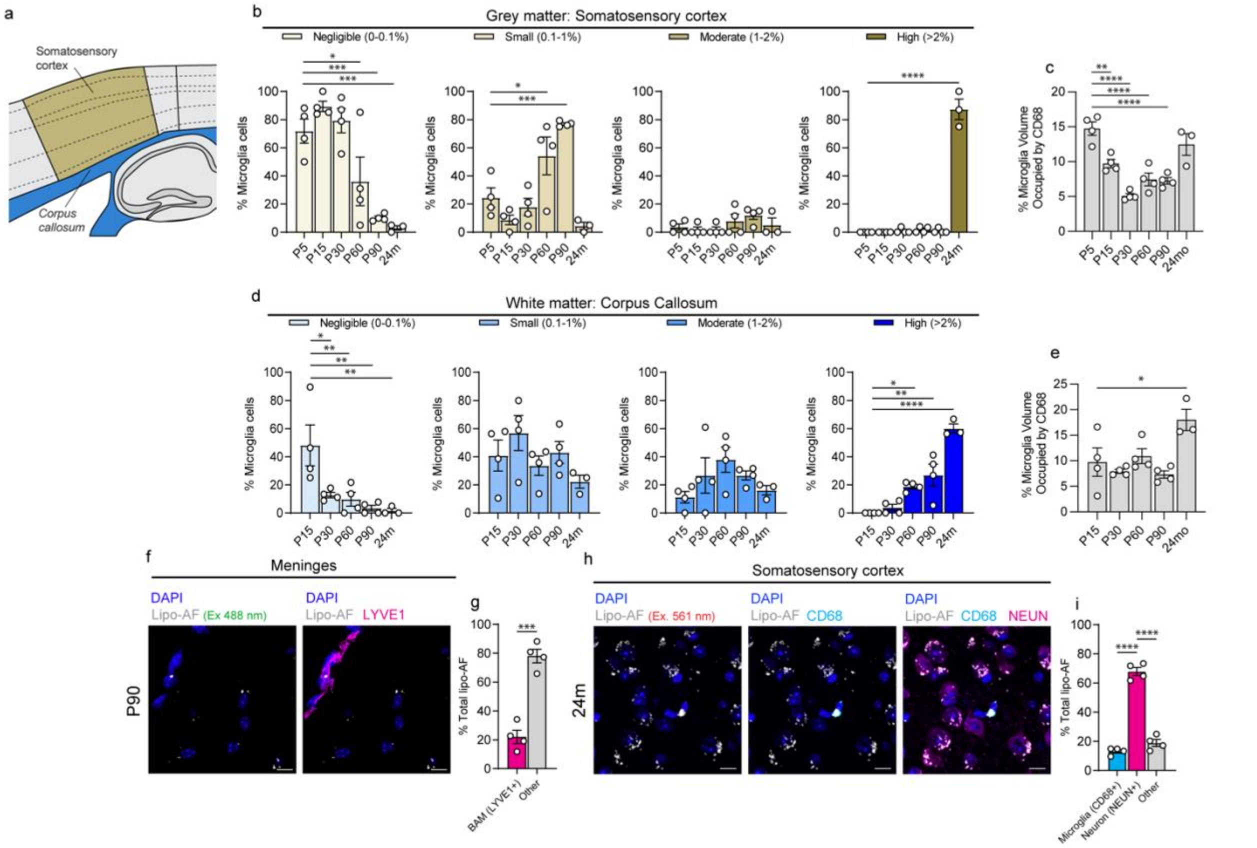


**Supplementary Figure 2. Lipo-AF increases with age.**

**a** Graphic illustrating brain regions analyzed. **b,d** Quantifications of each bin from Fig 2b or 2e. n = 3-4 mice (2M, 2F P5-P90; 2M, 1F 24m). One-way ANOVA with Dunnett's multiple comparisons test b (negligible, F = 15.50, df = 22, P5 vs P60 p = 0.0482, P5 vs P90 p = 0.0007, P5 vs 24m p = 0.0005; small, F = 15.51, df = 22, P5 vs P60 p = 0.0345, P5 vs P90 p = 0.0003; moderate, F = 1.577, df = 22; high, F = 193.2, df = 22, p<0.0001 ); *p<0.05,  ***p<0.001, ****p<0.0001. d (negligible, F = 6.68, df = 18, P15 vs P30 p = 0.0129, P15 vs P60 p = 0.0074, P15 vs P90 p = 0.0023, P15 vs 24m p = 0.0031; small, F = 1.667, df = 22,; moderate, F = 1.785, df = 22; high, F = 30.03, df = 18, P15 vs P60 = 0.0170, P15 vs P90 = 0.0011, P15 vs 24m p<0.0001 ); *p<0.05,  **p<0.01, ****p<0.0001. **c,e** The percentage of microglial volume occupied by CD68+ lysosomes. n = 3-4 mice (2M, 2F P5-P90; 2M, 1F 24m). One-way ANOVA with Dunnett's multiple comparisons test c (F = 19.58, df = 22, P5 vs. P15 p=0.0016, P5 vs. P30, P60, P90 p<0.0001); **p<0.01 ****p<0.0001. e (F = 5.40, df = 18, P15 vs. 24mo p=0.0190); **p<0.01. **f** Representative image of anti-LYVE1+ border associated meningeal macrophages and lipo-AF at P90. Scale bar = 10 µm. **g** The percentage of lipo-AF inside LYVE1+ border associated macrophages. n = 4 mice (3M, 1F). Two-tailed unpaired t-test (t = 8.503, df = 6, p=0.0001); ***p<0.001. **h** Representative image of anti-NeuN+ neurons, lipo-AF and anti-CD68+ microglial lysosomes in the 24m cortex. Scale bar = 10 µm. **i** Quantification of the percentage of lipo-AF inside NeuN+ neurons and CD68+ microglial lysosomes in 24m cortex. n = 4 mice (2M, 2F). One-way ANOVA with Tukey's multiple comparisons test (F = 177.2, df = 11, microglia vs. NeuN p<0.0001 and microglia vs. other p<0.0001); ****p<0.0001. All data are presented as a mean ± SEM.

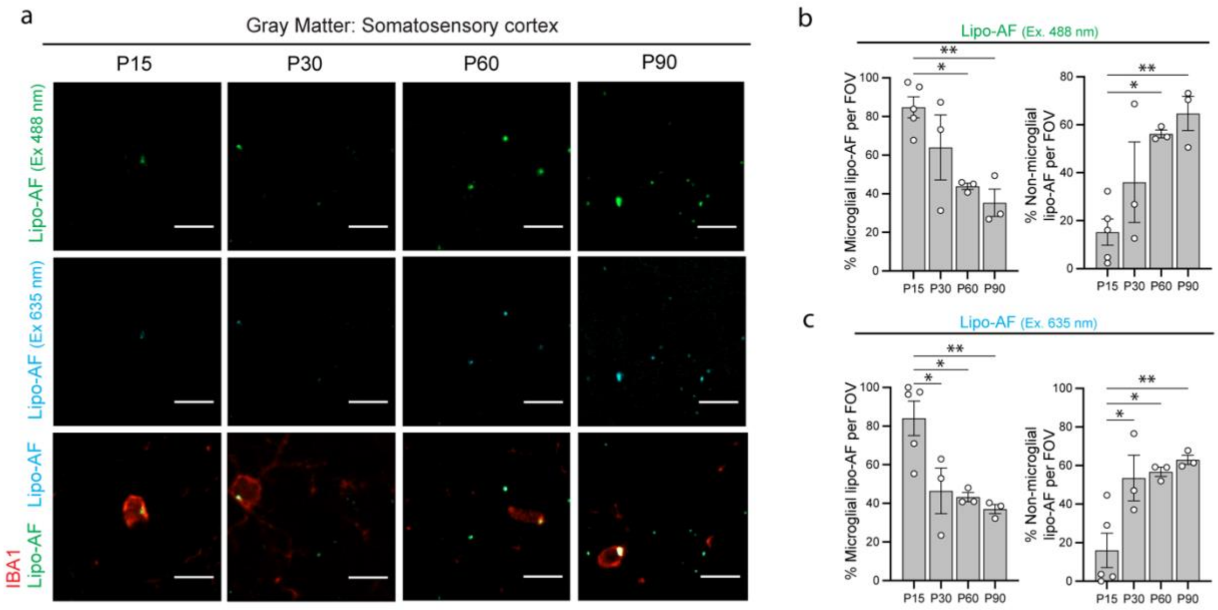


**Supplementary Figure 3. Early lipo-AF accumulation within microglia can be detected with multiple laser lines.**

**a** Representative images of lipo-AF excited with either the 488 nm or 635 nm laser and anti-IBA1+ immunolabelled microglia at different developmental time points. Scale bars = 10 µm. **b** Quantification of the percentage of total lipo-AF volume excited by a 488 nm laser per field of view outside and inside of IBA1+ microglia at different time points. Data are represented as mean ± SEM, n = 3-5 mice. One-way ANOVA with Dunnett's multiple comparisons test ($F = 7.273$, df = 13, P15 vs. P60 $p=0.0165$; P15 vs. P90 $p=0.0051$); *$p<0.05$ **$p<0.01$. **c** Quantification of the percentage of total lipo-AF volume excited by a 635 nm laser per field of view outside and inside of IBA1+ microglia at different time points. Data are represented as mean ± SEM, n = 3-5 mice. One-way ANOVA with Dunnett's multiple comparisons test ($F = 7.675$, df = 13, P15 vs. P30 $p=0.0231$; P15 vs. P60 $p=0.0147$; P15 vs. P90 $p=0.0061$); *$p<0.05$. (1M, 4F P15; 2M, 1F) for all other time points.

a b c

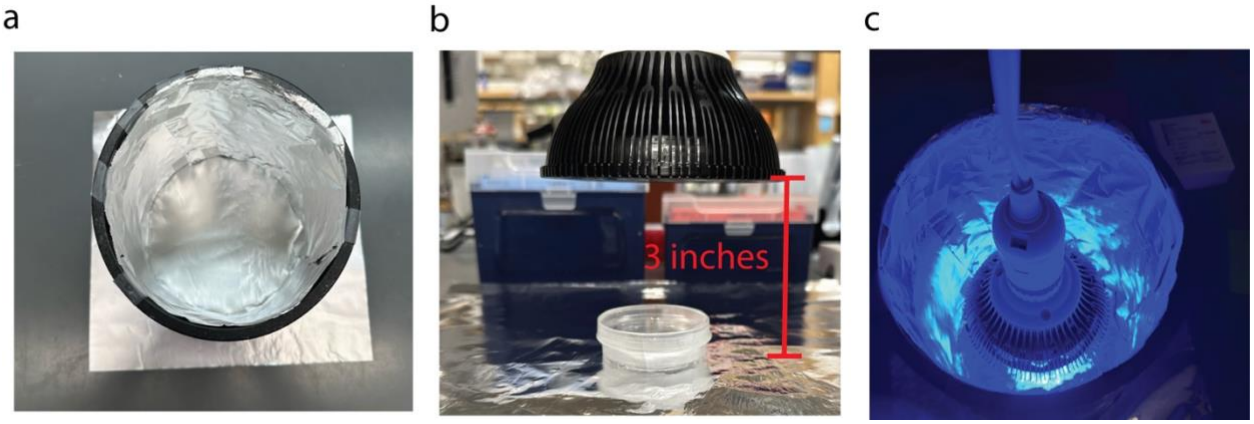


**Supplementary Figure 4. A cost effective and simple lab-made photobleacher.**
**a** Top-down view of lab-made photobleacher with an aluminum foil inside casing. **b** Horizontal view showing distance between the HiGrow LED bulb 450-460 nm and the tissue sample. **c** Top-down view of lab made photobleacher showing chamber when LED light has been turned on. Foil can be placed on top during photobleaching.

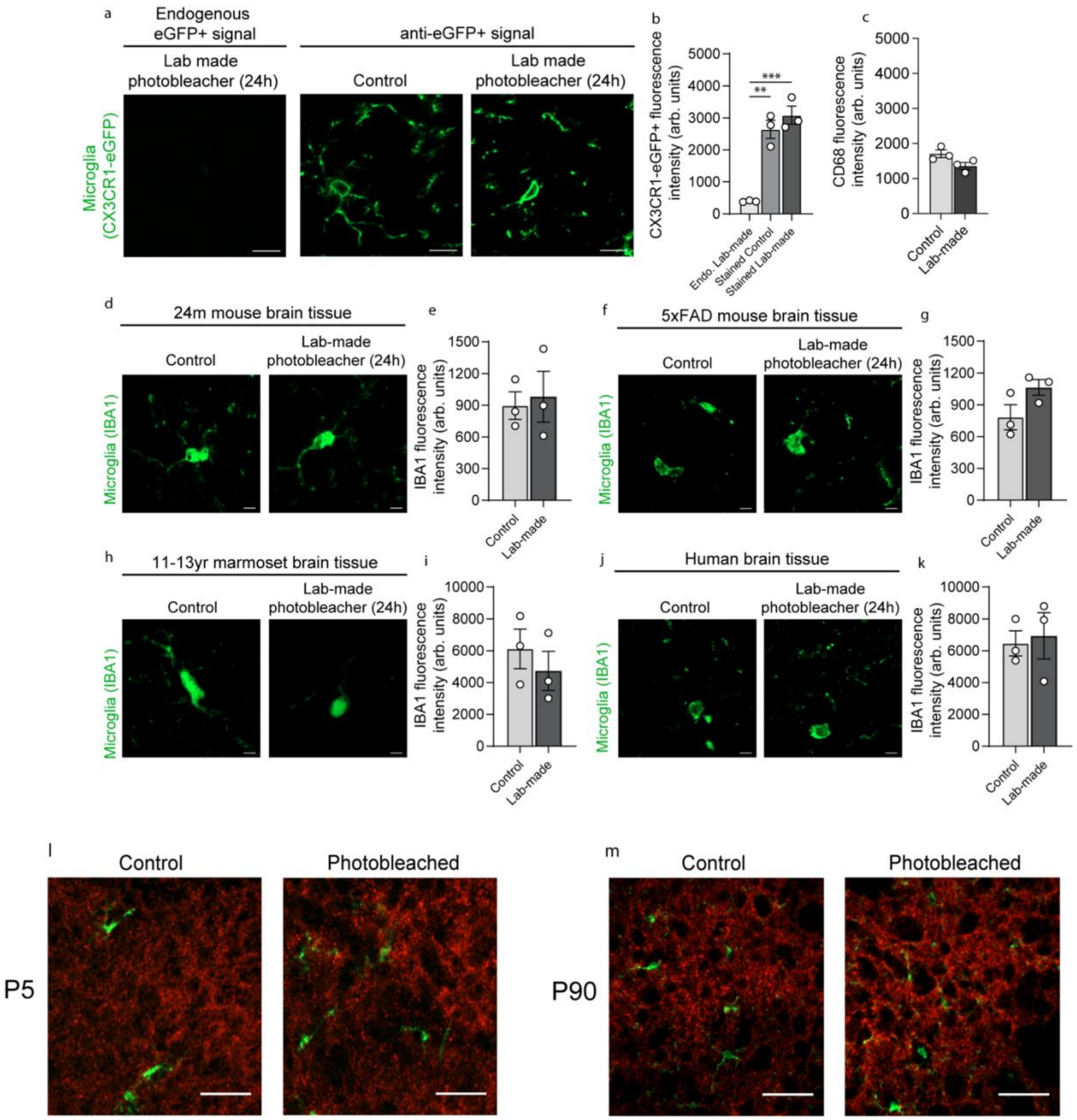


**Supplementary Figure 5. The lab-made photobleacher does not impact subsequently immunolabelling.**

**a** Representative images of CX3CR1-eGFP+ microglia from the mouse somatosensory cortex after photobleaching. Images show either endogenous fluorescence or anti-eGFP-immunolabelling signal. Scale bar = 10 µm. **b** Quantification of eGFP+ fluorescence intensity comparing photobleached endogenous (Endo) eGFP signal with anti-eGFP-immunolabelling signal with and without photobleaching. n = 3 mice (3M). One-way ANOVA with Tukey's multiple comparisons test (F = 40.69, df = 11, Endo lab-made vs. stained control p=0.0012; Endo lab-made vs. stained lab-made p=0.0004); **p<0.01 ***p<0.001. **c** Quantification of CD68 fluorescence with and without the lab-made photobleacher. n = 3 mice (3M). Paired two-tailed T test (t = 3.114, df = 2). **d** Representative images of

anti-IBA1 immunolabelled microglia in 24-month-old mouse somatosensory cortex. Scale bar = 5 µm. **e** Quantification of anti-IBA1 immunolabelled microglia fluorescence intensity before and after photobleaching in 24-month-old mouse somatosensory cortex. n = 3 mice (2M, 1F). Paired two-tailed T test (t = 0.3269, df = 2). **f** Representative images of anti-IBA1 immunolabelled microglia in 9-month-old 5xFAD mouse somatosensory cortex. Scale bar = 5 µm. **g** Quantification of anti-IBA1 immunolabelled microglia fluorescence intensity before and after photobleaching in 9-month-old 5xFAD mouse somatosensory cortex. n = 3 mice (1M, 2F). Paired two-tailed T test (t = 1.494, df = 2). **h,j** Representative images of anti-IBA1 immunolabelled microglia in FFPE 11-13-year-old marmoset brain (h) or in FFPE human MS brain (j). Scale bar = 5 µm. **i,k** Quantification of anti-IBA1 immunolabelled microglia fluorescence intensity before and after photobleaching in FFPE 11-13-year-old marmoset brain (i) or in FFPE human MS brain (k). n = 3 mice (2M, 1F for Marmoset; 3F for Human). i Paired two-tailed T test (t = 0.2126, df = 2). k Paired two-tailed T test (t = 3.260, df = 2). **l,m** Representative images at 20X of anti-IBA1+ immunolabelled microglia and anti-VGluT2+ synapses from layer IV of the P5 (g) or P90(h) somatosensory cortex with and without photobleaching. Scale bar = 50 µm. All data are represented as mean ± SEM.

**Supplementary Source Data**
