# Supplementary material for: Lipofuscin-like autofluorescence within microglia and its impact on studying microglial engulfment
Source: Nat Commun. 2023 Nov 3;14:7060. doi: 10.1038/s41467-023-42809-y (PMC10624656; doi:10.1038/s41467-023-42809-y)
Supplement: Supplementary file 3 — Reporting Summary [file 41467_2023_42809_MOESM3_ESM.pdf]

nature portfolio

Corresponding author(s): Dorothy Schafer
Last updated by author(s): Oct 13, 2023

Reporting Summary

Nature Portfolio wishes to improve the reproducibility of the work that we publish. This form provides structure for consistency and transparency in reporting. For further information on Nature Portfolio policies, see our Editorial Policies and the Editorial Policy Checklist.

Statistics

For all statistical analyses, confirm that the following items are present in the figure legend, table legend, main text, or Methods section.

n/a	Confirmed	
☐	☒	The exact sample size (*n*) for each experimental group/condition, given as a discrete number and unit of measurement
☐	☒	A statement on whether measurements were taken from distinct samples or whether the same sample was measured repeatedly
☐	☒	The statistical test(s) used AND whether they are one- or two-sided<br>*Only common tests should be described solely by name; describe more complex techniques in the Methods section.*
☐	☒	A description of all covariates tested
☐	☒	A description of any assumptions or corrections, such as tests of normality and adjustment for multiple comparisons
☐	☒	A full description of the statistical parameters including central tendency (e.g. means) or other basic estimates (e.g. regression coefficient) AND variation (e.g. standard deviation) or associated estimates of uncertainty (e.g. confidence intervals)
☐	☒	For null hypothesis testing, the test statistic (e.g. *F*, *t*, *r*) with confidence intervals, effect sizes, degrees of freedom and *P* value noted<br>*Give P values as exact values whenever suitable.*
☒	☐	For Bayesian analysis, information on the choice of priors and Markov chain Monte Carlo settings
☒	☐	For hierarchical and complex designs, identification of the appropriate level for tests and full reporting of outcomes
☒	☐	Estimates of effect sizes (e.g. Cohen's *d*, Pearson's *r*), indicating how they were calculated

Our web collection on statistics for biologists contains articles on many of the points above.

Software and code

Policy information about availability of computer code

Data collection	Zen Blue (2.3) Acquisition software was used for all experiments except Supp Figure 1a-b, where Zen (2.3) was used and Supp Fig. c-d where Zen Blue (3.2) was used
Data analysis	Data Analysis was performed using ImageJ Version 1.53c, Bitplane IMARIS version 9 and GraphPad PRISM 9.3.1

For manuscripts utilizing custom algorithms or software that are central to the research but not yet described in published literature, software must be made available to editors and reviewers. We strongly encourage code deposition in a community repository (e.g. GitHub). See the Nature Portfolio guidelines for submitting code & software for further information.

Data

Policy information about availability of data

All manuscripts must include a data availability statement. This statement should provide the following information, where applicable:

- Accession codes, unique identifiers, or web links for publicly available datasets
- A description of any restrictions on data availability
- For clinical datasets or third party data, please ensure that the statement adheres to our policy

A data availbility statement has been provided. Data for all graphs are included in the source data file. The files including images used for quantification is large and available upon request.

Research involving human participants, their data, or biological material

Policy information about studies with human participants or human data. See also policy information about sex, gender (identity/presentation), and sexual orientation and race, ethnicity and racism.

Reporting on sex and gender	Sex is reported. All subjects are female given the limited amount of tissue availability and the prevalence of MS in females.
Reporting on race, ethnicity, or other socially relevant groupings	TNU026 - Race: White. Ethnicity: Not Hispanic or Latino<br>TNU027 - Race: White. Ethnicity: Not Hispanic or Latino<br>TNU028 - Race: White. Ethnicity: Not Hispanic or Latino<br><br>This information was obtained from a NIH brain bank.
Population characteristics	The tissues were from a multiple sclerosis patient cohort with sex and other characteristics reported above. Age was betweem 60 and 77 years old.
Recruitment	n/a. This tissue was from a brain bank.
Ethics oversight	*Identify the organization(s) that approved the study protocol.*

Note that full information on the approval of the study protocol must also be provided in the manuscript.

Field-specific reporting

Please select the one below that is the best fit for your research. If you are not sure, read the appropriate sections before making your selection.

☒ Life sciences ☐ Behavioural & social sciences ☐ Ecological, evolutionary & environmental sciences

For a reference copy of the document with all sections, see nature.com/documents/nr-reporting-summary-flat.pdf

Life sciences study design

All studies must disclose on these points even when the disclosure is negative.

Sample size	Al sample sizes are provided. Sample sizes were chosen based on our previous work, which was sufficient to identify statistically significant biological effects.
Data exclusions	Outlier tests were performed on data sets and there was one data set where an outlier was identified and removed. (Fig. 4b-c) This is noted in the methods and in the source data.
Replication	Most experiments were performed and replicated by two independent experimenters (two co-first authors).
Randomization	All mice were randomly assigned to experimental groups.
Blinding	All experiments were blinded prior to analysis. Experiments were not blinded during collection as the age of mice is readily apparent and not possible to blind.

Reporting for specific materials, systems and methods

We require information from authors about some types of materials, experimental systems and methods used in many studies. Here, indicate whether each material, system or method listed is relevant to your study. If you are not sure if a list item applies to your research, read the appropriate section before selecting a response.

Materials & experimental systems

n/a	Involved in the study	
☐	☒	Antibodies
☒	☐	Eukaryotic cell lines
☒	☐	Palaeontology and archaeology
☐	☒	Animals and other organisms
☒	☐	Clinical data
☒	☐	Dual use research of concern
☒	☐	Plants

Methods

n/a	Involved in the study	
☒	☐	ChIP-seq
☒	☐	Flow cytometry
☒	☐	MRI-based neuroimaging

Antibodies

Antibodies used	Primary antibodies included: Rat mAb anti-CD68 (Abcam, ab955; 1:200), rabbit pAb anti-IBA1 (Wako Chemicals, 019-19741; 1:500), chicken mAb anti-IBA1 (Synaptic Systems, 234009; 1:500), rabbit pAb anti-P2RY12 (Anaspec, 55043A; 1:2000), guinea pig pAb anti-VGluT1 (Millipore, Ab5905; 1:1000), rabbit pAb anti-Homer1 (Synaptic Systems, 160003; 1:500), rabbit anti-lyve1 (Abcam, ab14917; 1:200), mouse anti-NeuN (Millipore, MAB377; 1:200), rabbit anti-GFP (Millipore, MAB3080p; 1:1000) and guinea pig pAb anti-VGluT2 (Millipore, Ab2251-I; 1:1000).<br><br>Secondary antibodies (all diluted at 1:1000) include goat anti-chicken IgY (H+L) Alexa-Fluor 488 (Life Technologies Scientific; A11039), goat anti-mouse IgG(H+L) Alexa-Fluor 488 (Life Technologies; A11029), goat anti-rabbit IgG (H+L) Alexa-Fluor 488 (Life Technologies; A11034), goat anti-guinea pig IgG (H+L) Alexa-Fluor 488 (Life Technologies; A11073), goat anti-rabbit IgG (H+L) Alexa-Fluor 594 (Life Technologies; A11012), goat anti-guinea pig IgG (H+L) Alexa-Fluor 594 (Life Technologies; A11076), goat anti-rabbit IgG (H+L) Alexa-Fluor 647 (Life Technologies; A21245), goat anti-guinea pig IgG (H+L) Alexa-Fluor 647 (Life Technologies; A21450), goat anti-rat IgG (H +L) Alexa-Fluor 647 (Life Technologies; A21247)
Validation	All primary and secondary antibodies have been extensively used in previous work. Validation statements for each antibody can be found on their respective websites.

Animals and other research organisms

Policy information about studies involving animals; ARRIVE guidelines recommended for reporting animal research, and Sex and Gender in Research

Laboratory animals	Postnatal day 5 (P5) to 24 month-old male and female C57Bl/6J mice (stock #000664) and Cx3cr1GFP/GFP mice (stock #005582) were obtained from Jackson Laboratories (Bar Harbor, ME). Adult common marmosets (Callithrix jacchus), both male and females between 11-13 years old, were obtained from the marmoset tissue library of translational neuroradiology section (TNS) at the NINDS.
Wild animals	N/A
Reporting on sex	Males and females were used for most, but not every, experiment involving animals. For most experiments where males and females were used, they were not in equal number f. Therefore, we did not perform separate analyses based on sex. In the source data and figure legends, we report the numbers of each sex for each experiment. Fig. 1b-c:1M and 2F; Fig. 1e-f: 3M and 1F; Fig. 1h-i 2F and 1M; Fig. 2: 2 M for each age and 1-2F for each age; Fig. 3: 3M WT and 2F and 1M 5xFAD; Fig. 4b,c,e: 2 M and 1-2F; Fig.4g,i: 3M; Fig. 5b-e: 3 M; Fig.5g-j,n: 2M and 2F; Fig. 5l: 2M and 1F; Fig. 6b,c,e,f: 2M and 1F.
Field-collected samples	N/A
Ethics oversight	All mouse data was acquired under the guidelines of the University of Massachusetts Chan Medical School Animal Care and Use Committees (IACUC) and NIH guidelines for ethics and proper use of animal welfare. All marmosets were housed and handled with the approval of the NINDS/NIDCD/NCCIH Animal Care and Use Committee (ACUC).

Note that full information on the approval of the study protocol must also be provided in the manuscript.

Plants

Seed stocks	*Report on the source of all seed stocks or other plant material used. If applicable, state the seed stock centre and catalogue number. If plant specimens were collected from the field, describe the collection location, date and sampling procedures.*
Novel plant genotypes	*Describe the methods by which all novel plant genotypes were produced. This includes those generated by transgenic approaches, gene editing, chemical/radiation-based mutagenesis and hybridization. For transgenic lines, describe the transformation method, the number of independent lines analyzed and the generation upon which experiments were performed. For gene-edited lines, describe the editor used, the endogenous sequence targeted for editing, the targeting guide RNA sequence (if applicable) and how the editor was applied.*
Authentication	*Describe any authentication procedures for each seed stock used or novel genotype generated. Describe any experiments used to assess the effect of a mutation and, where applicable, how potential secondary effects (e.g. second site T-DNA insertions, mosiacism, off-target gene editing) were examined.*
